# The impact of *Disrupted-in-Schizophrenia 1* (*DISC1*) on the dopaminergic system: a systematic review

**DOI:** 10.1038/tp.2016.282

**Published:** 2017-01-31

**Authors:** T Dahoun, S V Trossbach, N J Brandon, C Korth, O D Howes

**Affiliations:** 1 Psychiatric Imaging Group MRC London Institute of Medical Sciences, Hammersmith Hospital, London, UK; 2 Institute of Clinical Sciences, Faculty of Medicine, Imperial College, Hammersmith Hospital, London, UK; 3 Department of the Institute of Clinical Sciences, Psychiatric Imaging Group, MRC London Institute of Medical Sciences (LMS), Imperial College-Hammersmith Hospital Campus, London, UK; 4 Department of Neuropathology, Medical Faculty, Heinrich Heine University Düsseldorf, Düsseldorf, Germany; 5 AstraZeneca Neuroscience, Innovative Medicines and Early Development Biotech Unit, R&D Boston, Waltham, MA, USA; 6 Department of Psychosis Studies, Institute of Psychiatry, Neurology and Neuroscience (IoPPN), King's College London, London, UK

## Abstract

*Disrupted-in-Schizophrenia 1* (*DISC1*) is a gene known as a risk factor for mental illnesses possibly associated with dopamine impairments. DISC1 is a scaffold protein interacting with proteins involved in the dopamine system. Here we summarise the impact of *DISC1* disruption on the dopamine system in animal models, considering its effects on presynaptic dopaminergic function (tyrosine hydroxylase levels, dopamine transporter levels, dopamine levels at baseline and after amphetamine administration) and postsynaptic dopaminergic function (dopamine D1 and D2 receptor levels, dopamine receptor-binding potential and locomotor activity after amphetamine administration). Our findings show that many but not all *DISC1* models display (1) increased locomotion after amphetamine administration, (2) increased dopamine levels after amphetamine administration in the nucleus accumbens, and (3) inconsistent basal dopamine levels, dopamine receptor levels and binding potentials. There is also limited evidence for decreased tyrosine hydroxylase levels in the frontal cortex and increased dopamine transporter levels in the striatum but not nucleus accumbens, but these conclusions warrant further replication. The main dopaminergic findings are seen across different *DISC1* models, providing convergent evidence that *DISC1* has a role in regulating dopaminergic function. These results implicate dopaminergic dysregulation as a mechanism underlying the increased rate of schizophrenia seen in *DISC1* variant carriers, and provide insights into how DISC1, and potentially DISC1-interacting proteins such as AKT and GSK-3, could be used as novel therapeutic targets for schizophrenia.

## Introduction

The *Disrupted-in-Schizophrenia 1* (*DISC1*) gene was originally discovered at the breakpoint of a balanced translocation t(1;11) (q42;q14.3) in a Scottish family and later identified in a North American family with high rates of schizophrenia.^1, 2, 3, 4^ Since then, preclinical models have shown that *DISC1* mutant animals exhibit behavioural, neurostructural and neurochemical features relevant to schizophrenia, ^5, 6^ although its significance for the human disease has been debated.^7, 8, 9^ DISC1 is described as a scaffold protein with multiple interactors involved in a wide range of cellular processes including neurotransmitter signalling.^10, 11^ In particular, DISC1 is known to interact with several proteins involved in dopamine signalling including fasciculation and elongation protein zeta 1, phosphodiesterase 4D9 and phosphodiesterase 4B, serine/threonine protein kinase Akt and glycogen synthase kinase-3 (GSK-3)^12, 13, 14, 15, 16^ as well as synaptic interactors such as kalirin-7 and the Traf2, Nck-interacting kinase,^17, 18^ and the microtubule/centrosomal proteins pericentriolar material 1 and Bardet–Biedl syndrome protein.^19, 20^ These multiple interactions have highlighted the potential of DISC1 as a therapeutic target.^21, 22, 23^

The neurotransmitter dopamine is widely thought to have a central role in the aetiology of psychotic disorders.^24, 25, 26^ The dopamine hypothesis of schizophrenia was initially based on the findings that the affinity of antipsychotic medications for dopamine receptors is closely related to their clinical potency,^27, 28, 29^ and that drugs that increase dopamine levels provoke psychotic symptoms in healthy people.^30, 31^ Molecular imaging studies since then have shown increased presynaptic dopamine synthesis capacity and release in schizophrenia^32, 33, 34, 35^ and in subjects with prodromal symptoms of schizophrenia.^36, 37, 38, 39^ Alterations in dopamine D1 and D2/3 receptors, tyrosine hydroxylase (TH) levels and baseline synaptic dopamine levels in schizophrenia have also been reported,^40, 41^ although with some inconsistency.^42^

These findings highlight why dopaminergic dysfunction has a pivotal role in schizophrenia. In view of this, we sought to review the evidence from animal models that *DISC1* pathway alterations may impact on dopaminergic function, as it has not been comprehensively synthesised before. The aim of our review was therefore to summarise the impact of DISC1 on TH levels, dopamine transporter (DAT) levels, basal dopamine levels and after amphetamine administration, dopamine D2 receptor-binding potential (BP), dopamine D1 (D1R) and D2 receptor (D2R) levels, and locomotor activity after amphetamine administration for dopamine-related behaviour.^43^ We selected publications citing data collection in the midbrain, as this is the location of the majority of dopaminergic neuron cell bodies in the brain, and the frontal cortex, hippocampus and striatum as these are the target sites of the main dopaminergic pathways relevant to psychiatric disorders.^44, 45^

## Materials and methods

### Selection of studies

The entire PubMed database was searched to select publications. Studies were screened based on the terms (‘Disrupted-in-Schizophrenia-1' OR ‘*DISC1*') AND (‘dopamine' OR ‘tyrosine hydroxylase' OR ‘dopamine receptor' OR ‘DAT' OR ‘amphetamine' OR ‘behavioral alterations' OR ‘locomotor activity' OR ‘Positron Emission Tomography' OR ‘PET' OR ‘Single Photon Emission Computed Tomography' OR ‘SPECT'). Only articles meeting the following criteria were included: (1) original studies; (2) English language; (3) peer-review journals; (4) findings reporting TH levels, DAT levels, basal dopamine levels and/or dopamine levels after amphetamine administration, and/or dopamine receptor-binding potential, dopamine receptor levels and/or locomotion after amphetamine administration in a *DISC1* model compared with a control group; and (5) in the frontal cortex, striatum, nucleus accumbens, midbrain and/or hippocampus, as these regions are major target sites of dopaminergic projections in the brain and are thought to be involved in the pathophysiology of schizophrenia.^44, 45^ The *DISC1* models were selected based on gene mutation in *DISC1* or alteration in the quantitative expression of DISC1 protein. Method and results sections of the eligible articles were screened to identify the measures of interest listed above.

### Data extraction

The main outcome measures were the differences between the *DISC1* models and controls in (1) TH levels; (2) DAT levels; (3) basal dopamine levels; (4) dopamine levels after amphetamine administration; (5) dopamine receptor-binding potential; (6) D1R and D2R levels; and (7) locomotion after amphetamine administration. In addition, the following data were extracted: (8) authors; (9) year of publication; (10) the *DISC1* model; (11) samples size; and (12) methods. The data were extracted by TD and checked by SVT. Findings related to the nucleus accumbens and olfactory tubercle were merged as both being part of the ventral striatum.^46^

## Results

**Figure 1:**
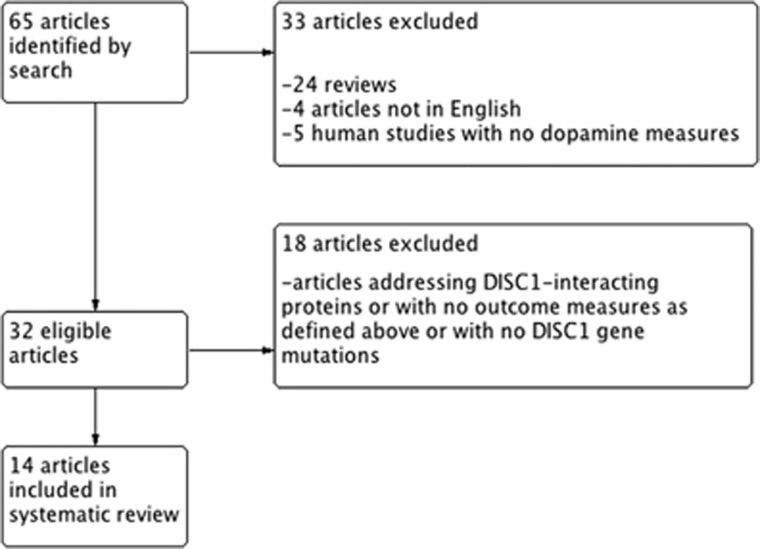
Flow chart of identification, exclusion and inclusion of eligible studies. DISC1, disrupted-in-schizophrenia 1.

Fifty-one studies were excluded from a total of 65 studies screened (Figure 1). Fourteen studies were included of which two were of TH levels, three of DAT levels, nine of basal dopamine levels, six of induced dopamine release, four of dopamine receptor BP, four studies of D1R levels, four studies of D2R levels and thirteen of locomotion after amphetamine administration. Table 1 summarises all studies including the *DISC1* model used, sample sizes and methods. It should be noted that we were not able to find evidence that dopaminergic function had been investigated in more recently disclosed *DISC1* models, for example.^47, 48^

### *DISC1* models

Five types of *DISC1* models were identified across the studies as follows: (1) transgenic expression of truncated human *Disc1* protein with dominant-negative (DN) effect; (2) *DISC1* haploinsufficiency/silencing; (3) full-length human *DISC1* overexpression; (4) artificial *Disc1* mutation; and (5) wild-type model (Table 1). Data on locomotion after amphetamine administration from Su *et al.*^62^ were included despite the absence of a direct comparison between mutant and wild-type mice as they showed a functional relationship between Disc1 and the dopamine receptor. Both genotype effects (wild type versus transgenic) and genotype effect in a stress condition (isolated wild type versus isolated transgenics) were included from Niwa *et al.*^51^

### TH levels

**Figure 2:**
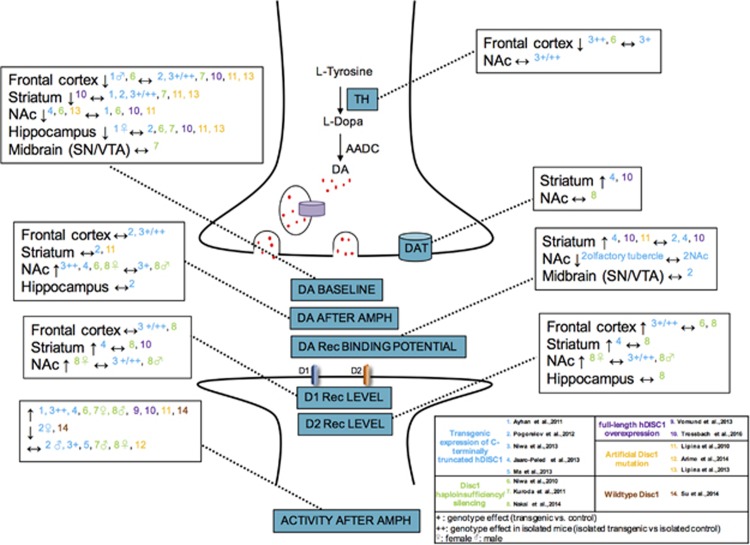
The impact of *DISC1* models on the dopamine system. AMPH, amphetamine; DA, dopamine; DAT, dopamine transporter; NAc, nucleus accumbens; SN, substantia nigra; TH, tyrosine hydroxylase; VTA, ventral tegmental area.

Two studies investigated TH levels in the *hDISC1* and the *Disc1* RNA interference (RNAi)/silencing models compared with controls.^51, 54^ These studies showed reduced TH levels in frontal cortical regions in isolated *hDISC1* mice compared with isolated controls^51^ and in the *Disc1* RNAi/silencing model compared with controls^54^ (Figure 2 and Table 3).

One study showed no significant changes in TH levels between *hDISC1* and controls, and between isolated *hDISC1* mice and isolated controls in the nucleus accumbens.^51^

### DAT levels

Three studies investigated DAT levels in the *DISC1* model compared with controls.^52, 56, 58^ Two studies found increased DAT levels in the striatum of DN homozygous line 37 mice and tg*DISC1* rats compared with controls Tables 2 and 3.^52, 58^

One study found no significant difference between the *Disc1*Δ2–3 mice compared with controls in the nucleus accumbens.^56^

### Basal dopamine levels

Nine studies investigated basal dopamine levels in *DISC1* models compared with controls.^49, 50, 51, 52, 54, 56, 58, 59, 61^ *In vivo* microdialysis and *post-mortem* high-performance liquid chromatography with electrochemical detection (HPLC-ED) were used, measuring extracellular and total dopamine levels, respectively.

Eight studies investigated basal dopamine levels in the frontal cortex/mPFC, six using HPLC-ED^49, 50, 55, 58, 59, 61^ and two using both *post-mortem* HPLC-ED and microdialysis.^51, 54^ One of the two studies using microdialysis showed decreased basal dopamine levels in the *Disc1* RNAi/silencing model compared to controls,^54^ whereas the other study found no significant changes between the *hDISC1* mice and controls and the isolated *hDISC1* mice and isolated controls.^51^ For HPLC-ED, decreased basal dopamine levels were found at postnatal day 56 in the *Disc1* RNAi/silencing model,^54^ and in males from the prenatal *hDISC1* expression group (until embryonic day 17), the postnatal expression group (from embryonic day 17 on) groups and the pre- and postnatal *hDISC1* expression (entire life) compared with controls.^49^ No significant differences were reported in the other studies.^50, 51, 55, 58, 59, 61^

Six studies investigated basal dopamine levels in the striatum using HPLC-ED^49, 50, 51, 55, 58, 61^ and one using both HPLC-ED and microdialysis.^59^ One study found decreased total dopamine levels in the full-length *DISC1*-overexpressing rat model compared with controls in the dorsal striatum.^58^

Six studies investigated basal dopamine levels in the nucleus accumbens, one using *in vivo* microdialysis,^52^ four using HPLC-ED^49, 58, 59, 61^ and one using both techniques.^54^ Two studies using *in vivo* microdialysis showed decreased basal dopamine levels in the *Disc1* RNAi/silencing model compared with controls^54^ and the *hDISC1* heterozygous line 10 and 37 mice compared with controls.^52^ One study using HPLC-ED showed significant decreased basal dopamine levels in L100P ENU-generated missense mutation mice,^61^ whereas the others found no significant differences.^49, 54, 58, 59^

One study investigated basal dopamine levels in the midbrain and found no significant difference between the *Disc1*Δ2–3 haploinsufficiency model and controls.^55^

Seven studies investigated basal dopamine levels in the hippocampus using HPLC-ED.^49, 50, 54, 55, 58, 59, 61^ One study found decreased dopamine levels in females in the postnatal *hDISC1* expression group compared with prenatal expression only and controls.^49^ The other studies found no significant differences.^50, 54, 55, 58, 59, 61^

### Induced dopamine release

All the studies induced dopamine release by administrating amphetamine-related drugs. Two studies investigated induced dopamine release in the frontal cortex and found no significant differences, one using microdialysis^51^ and one using HPLC-ED.^50^

One study investigated induced dopamine release in the striatum using *in vivo* microdialysis^59^ and one study using HPLC-ED,^50^ both reporting no significant differences.

Four studies investigated induced dopamine release in the nucleus accumbens using microdialysis.^51, 52, 54, 56^ The four studies found significantly increased dopamine release. This was in the *DISC1* knockdown compared with controls,^54^ in isolated *hDISC1* compared with isolated controls,^51^ in heterozygous line 10 and 37 compared with controls ^52^ and female but not male *Disc1*Δ2–3 mice compared with controls.^56^

One study investigated induced dopamine release in the hippocampus using HPLC-ED and found no significant difference between the *hDISC1* and controls.^50^

### Dopamine D1 receptor

Two studies investigated D1R levels in the frontal cortex and found no significant differences between the *hDISC1* and controls, and the *Disc1*Δ2–3 haploinsufficiency model and controls.^51, 56^

Three studies investigated D1R levels in the striatum.^52, 56, 58^ One study found increased levels in the *hDISC1* model compared with controls,^52^ whereas the others found no significant differences.^56, 58^

Two studies investigated D1R levels in the nucleus accumbens.^51, 56^ One study found significant increased D1R levels in female and no significant changes in male and mixed *Disc1*Δ2–3 groups,^56^ whereas the other showed no significant differences.^51^

### Dopamine D2 receptor

Three studies investigated D2R levels in the frontal cortex.^51, 54, 56^ One study found significant increased D2R levels in the *hDISC1* mice compared with controls and isolated *hDISC1* mice compared with isolated controls^51^ and the two other studies found no significant differences between the *Disc1* RNAi/silencing/haploinsufficiency models and controls.^54, 56^

Two studies investigated D2R levels in the striatum.^52, 56^ The *hDISC1* mice showed significant increased D2R levels,^52^ whereas the other showed no significant differences between the *Disc1*Δ2–3 models and controls.^56^

Four studies investigated dopamine receptor-binding potential in the striatum.^50, 52, 58, 59^ The dopamine D2 receptor is known to exist in two interconverting states, a low-affinity (μm) and a high-affinity (nm) state.^63^ Lipina *et al.*^59^ and Trossbach *et al.*^58^ found a significant increase in dopamine D2 high-affinity receptor levels using [^3^H]domperidone binding challenged with dopamine, but Trossbach *et al.* found no difference in [^3^H]raclopride binding by autoradiography. As raclopride does not distinguish low from high affinity or D2 from D3 receptors, taken together, these studies are consistent with a shift to the high-affinity state without a change in total D2/3 receptor levels. Jaaro-Peled *et al.*^52^ found significantly increased binding potential of D2/3 receptor availability in the striatum using [^11^C]raclopride PET and significantly increased levels of D2/3R in the medial part of the right rostral striatum using [^3^H]spiperone autoradiography but no significant differences in D2/3 levels in the total right rostral striatum and the lateral part of the right striatum in the *hDISC1* compared with controls. Pogorelov *et al.*^50^ found no significant difference in the rostral part of the striatum using [^11^C]raclopride autoradiography in the *hDISC1* mice compared with controls.

Two studies investigated D2R levels in the nucleus accumbens.^51, 56^ One study found significantly increased D2R levels in female but not male and mixed *Disc1*Δ2–3 groups,^56^ whereas the other showed no significant differences.^51^

One study investigated D2/3R-binding potential in the nucleus accumbens using [^11^C]raclopride autoradiography PET and found no significant differences in the nucleus accumbens but significantly decreased levels in the right olfactory tubercle of female *hDISC1* mice compared with controls.^50^ They used the same approach to investigate D2/3R-binding potential using [^11^C]raclopride autoradiography in the midbrain (substantia nigra/VTA) and found no significant difference between the *hDISC1* and controls.^50^

One study investigated D2R levels in the hippocampus and found no significant difference between the *Disc1*Δ2–3 haploinsufficiency and controls.^56^

### Locomotion after amphetamine administration

Thirteen studies investigated locomotion after amphetamine administration.^49, 50, 51, 52, 53, 54, 55, 56, 57, 58, 59, 60, 62^ Ten studies found increased locomotion after amphetamine administration in the *DISC1* models compared with control animals, in the pre- and postnatal *hDISC1* expression groups,^49^ *hDISC1* mice,^52^ the *Disc1* RNAi/silencing model,^54^ female but not male *Disc1*Δ2–3 mice,^55^ male but not female *Disc1*Δ2–3 mice,^56^ full-length *hDISC1*-overexpressing rats,^57, 58^ *Disc1*-L100P mice,^59^ isolated *hDISC1* mice compared with isolated controls^51^ and wild-type *Disc1* mice with no *DISC1*–D2R disruption.^62^ Two studies found decreased locomotion after amphetamine administration, in female but not male *hDISC1* mice after escalating dose of methamphetamine treatment compared with controls in Pogorelov *et al.*^50^ and wild-type mice with *Disc1*–D2R disruption in Su *et al.*^62^ No significant changes were found in the *hDISC1* mice and *Disc1*-L100P/L100P mice compared with controls in three studies.^51, 53, 60^

## Discussion

The main findings show that compared with controls, the *DISC1* models exhibit reasonably consistent (1) increased locomotion after amphetamine administration (2) increased dopamine levels after amphetamine administration in the nucleus accumbens but (3) inconsistent alterations in basal dopamine levels and dopamine receptor levels and binding potentials. These findings extend other studies showing increased methamphetamine-induced dopamine release in the nucleus accumbens and locomotor hyperactivity in mice lacking DISC1-interacting proteins, such as fasciculation and elongation protein zeta 1^64^ and PDE4,^65^ to indicate that the *DISC1* pathway affects specific aspects of dopaminergic function.

### Limitations

The findings presented in this systematic review must be considered in the light of the following limitations. First, the number of studies was modest for some aspects of dopaminergic function, such as transporter levels, and some regions. This limits the conclusions that can be drawn about these aspects, and highlights the needs for further studies. Second, the studies used a heterogeneous set of *DISC1* models (Table 1), which could contribute to variability in results. Third, the evidence comes from a relatively small number of research groups. Thus, replication would be useful to determine generalisability. And fourth, alterations in other neurotransmitter system such as noradrenaline might also contribute to the locomotor hyperactivity phenotype observed. However, several reports indicate that locomotor hyperactivity after amphetamine is specifically mediated through dopamine and not noradrenergic transmission in the nucleus accumbens.^66, 67, 68^

### Potential mechanisms underlying locomotor hyperactivity

The majority of the *DISC1* models used showed locomotor hyperactivity following amphetamine challenge. This shows a relatively conserved phenotype of the *DISC1* models that might be explained by (1) the presynaptic effects of *DISC1* on dopamine release in the nucleus accumbens or (2) a direct impact of the *DISC1* models on postsynaptic dopaminergic signal transduction, such as the protein serine/threonine protein kinase (Akt)–glycogen synthase kinase-3 (GSK-3) pathway. In support of the first hypothesis, the nucleus accumbens is thought to have an important role in regulating locomotor activity.^69, 70^ Local administration of dopamine and amphetamine has been shown to induce hyperactivity similar to systemic administration,^66, 70, 71, 72^ and our review has identified reasonably consistent evidence that *DISC1* models are associated with greater dopamine release to amphetamine. With regards to the second hypothesis, Akt and GSK-3 are two proteins regulated by DISC1 with respectively indirect and direct interactions.^15, 16, 73^ The Akt–GSK-3 pathway modulates dopamine neurotransmission and amphetamine-mediated locomotor activity.^74, 75, 76^ Amphetamine/methamphetamine-induced dopamine release decreases Akt activation (phosphorylation state^77^), which activates GSK-3 by dephosphorylating the Serine 9 site^78^ to modulate dopamine-dependent behaviours.^74^ Although Disc1 wild-type protein decreases Akt and GSK-3 activation,^15, 73, 79^ the impact of mutant DISC1 on Akt and GSK-3 is less clear. Evidence shows increased and decreased Akt activation in *DISC1* knockdown,^15, 62, 80^ no effects on Akt and GSK-3 levels in *hDISC1* mice^50^ and consistently increased GSK-3 activation in *DISC1* knockdown and *Disc1* point mutation Q31L.^62, 81, 82^ Interestingly, mice overexpressing GSK-3 develop locomotor hyperactivity,^83^ *GSK-3* knockdown mice express reduced locomotor activity^84^ and administration of GSK-3 inhibitor decreases amphetamine-induced hyperactivity.^85^

### Potential mechanisms underlying increased dopamine release to amphetamine

The studies reporting increased dopamine levels following amphetamine administration in the nucleus accumbens used a *Disc1*Δ2–3 haploinsufficiency,^56^ a DN *hDISC1* model in combination with adolescent isolation stress,^51^ a transient knockdown in prefrontal cortex ^54^ and a DN *hDISC1* model targeting specifically pyramidal neurons of the cortex and hippocampus.^52^ This raises the questions of (1) the time course of changes in dopamine and whether there are developmental periods that are particularly vulnerable to DISC1 alterations, (2) the brain regions minimally required to lead to increased dopamine release to amphetamine, and in particular, the role of the cortical regions in regulating the nucleus accumbens dopamine levels.

With regards to the first point, recent studies suggest that DISC1 alterations interact with stress to impact on dopaminergic neurons during adolescence.^51, 86^ These findings are in line with evidence showing that adolescence is a critical time life for the development of psychotic disorders including schizophrenia.^87^ With regards to the second point, a possible mechanism underlying increased dopamine levels in the nucleus accumbens could be a reduction in cortical parvalbumin-positive interneurons. Supporting this, studies have shown a decreased number of parvalbumin-positive interneurons in the cortex of DN *DISC1* models.^49, 88, 89, 90^ Parvalbumin-positive interneurons are GABAergic inhibitory neurons thought to regulate the dopaminergic activity in the nucleus accumbens and to have a role in schizophrenia through the modulation of cortical glutamate excitatory pyramidal neurons.^91, 92, 93^ Finally, the specific localisation of the findings in the nucleus accumbens might be related to an increased sensitivity of this region to stimulants, as it has been shown to release more dopamine following amphetamine administration compared with other striatal subdivisions.^94^

### Inconsistent basal dopamine levels and dopamine receptor-binding potential and levels

We summarise here a series of inconsistent findings on basal dopamine levels and dopamine receptor-binding potentials and levels in the frontal cortex, striatum, nucleus accumbens and hippocampus. These findings might be due the heterogeneity of the *DISC1* models used (Table 1). Among these, only the short interfering RNA knockdown or knockout models should have loss of function phenotypes whereas all others could have either loss of function, or gain of function, or combined phenotypes at the same time. However, no more consistency is observed when looking only at the loss of function models. It should also be noted that the tg*DISC1* rat was conceived as a model for protein pathology related to DISC1 rather than a model for mutant *DISC1*.^58, 95^ Another possible explanation could be that these are adaptive changes not always seen following the core dopamine release alteration.

### Implications

The effects of DISC1 on dopamine release and the behavioural effects of amphetamine are in line with evidence showing increased amphetamine-induced dopamine release in schizophrenia, and that this positively correlates with amphetamine-induced positive psychotic symptoms.^34, 35, 96, 97^ The absence of clear receptor changes is also consistent with the lack of changes in dopamine D2/3 receptors alterations seen in a meta-analysis of *in vivo* findings in schizophrenia.^98^ However, the inconsistent findings in striatal basal dopamine levels do not agree with the *in vivo* evidence showing increased basal dopamine levels in schizophrenia.^33, 99^ Taken together, these findings indicate that *DISC1* alterations may increase the risk of schizophrenia by dysregulating the presynaptic regulation of dopamine but they do not result in the full dopaminergic phenotype, suggesting other factors must interact with DISC1. Stress is one likely candidate factor^100^ that has been shown increase dopamine release in psychosis.^38^

It should be noted that *DISC1* is also associated with affective disorders including major depression.^101, 102^ The implications of the findings for this association remains unclear, as human PET studies have shown decreased dopamine synthesis capacity in patients with major depression particularly in individuals with reduced affect or psychomotor slowing symptoms^103, 104, 105^ and some endophenotypes such as anhedonia are thought to be underlined by dopamine function.^106, 107^

Our conclusions drawn from the preclinical research reviewed here may have interesting implications for clinical research and hence translational value at pointing to the necessity of identifying a biomarker to identify illness subtypes related to DISC1 dysfunction, to guide treatment choice and as a lead for the development of novel therapies. Determining whether DISC1 function is aberrant in a given individual could be a useful to subtype patients. Given that aberrant DISC1 function modulates aspects of dopaminergic function, this may help identify patients who may be responsive to drugs that act on the dopaminergic system, in line with emerging evidence on dopaminergic and non-dopaminergic subtypes of schizophrenia.^108^ What directions could the search for identifying biomarkers for aberrant DISC1 function take? Screening for DISC1 polymorphisms may be one way to assess this as some polymorphisms have been associated with different neuronal functions and with treatment-resistant schizophrenia.^109, 110, 111^ As it has been demonstrated that single-nucleotide polymorphisms of DISC1-interacting genes are overrepresented in schizophrenia,^112^ the use of a DISC1-interactome polygenic risk score might also be a complementary approach to stratify the risk associated with a specific signalling pathway or response to treatment. However, it should be recognised that genetic diagnostics alone may not provide sufficient information because DISC1 levels also depend on other factors, for example, BACE1-dependent cleavage of neuregulin 1.^113^ Large cohort studies of patients are needed to determine whether *DISC1* polymorphisms and/or DISC1 protein levels in peripheral cells do identify subsets of patients with distinct illness characteristics or treatment response.^114^ This may require the combinatorial analysis of blood-based, imaging and/or neurophysiological factors, to both identify those patients with both aberrant DISC1 and neuronal function. Another key implication is that understanding how *DISC1* alterations lead to dopamine dysregulation could identify new treatment approaches to address the dopamine dysfunction seen in schizophrenia and people at risk of schizophrenia in a broader sense. Pharmacological targeting of aberrant DISC1 function may be able to correct dopamine dysfunction without directly interfering with dopamine receptors themselves, providing an alternative to existing antipsychotic drugs, which are all D2/3 receptor blockers. In that sense, clinical development of diagnostics and pharmacotherapy of DISC1-related disorders may go hand in hand^95^ to support the development of precision medicine in psychiatry.

### Future directions

We identify four key lines of direction for future studies based on the findings: first, as results to date come from a relative small number of studies, it would be useful to investigate dopamine function in *DISC1* models recently developed.^47, 48^ Second, the mechanism by which *DISC1* leads to increased dopamine release to amphetamine needs further investigation, in particular to determine whether this could be mediated by disinhibition of parvalbumin-positive interneurons or the Akt–GSK-3 pathway. Interestingly, a *DISC1* model has been recently developed with selective knockdown of interneuronal DISC1 in parvalbumin neurons,^47^ which might provide insightful knowledge on the mechanisms linking *DISC1* and dopamine regulations. In that context, it is also remarkable that *DISC1* as a single factor is able to both regulate dopamine neuroanatomy as well as parvalbumin-positive interneuron placement in cortical layers.^115^ Third, elevated dopamine synthesis capacity is the other aspect of presynaptic dopamine dysregulation widely linked to schizophrenia and people at risk of schizophrenia.^36, 37, 116^ Thus, future work should test if *DISC1* alterations affect this aspect of presynaptic dopamine function in humans. Fourth, it would be useful to examine further the impact of environmental stress on dopamine release and dopamine levels in *DISC1* models as proposed by some authors.^51, 117^

## Conclusions

Compared with controls, the majority of the *DISC1* models but not all exhibits (1) increased locomotion after amphetamine administration and (2) increased dopamine levels after amphetamine administration in the nucleus accumbens but (3) inconsistent basal dopamine levels and dopamine receptor levels and binding potentials. This suggests that presynaptic dopamine dysregulation is a potential mechanism for the increased rates of psychotic disorders seen in the original *DISC1* families and *DISC1* variant carriers, and identifies a number of potential therapeutic targets for treating or even preventing schizophrenia based on the *DISC1* pathway.

## Figures and Tables

**Table 1:** *DISC1* models with available dopamine-related data

*DISC1 model category*	*Authors*^ref.^		*Functional impact on DISC1*	*Method*	*Rodent strain*	*Promoter*	*Affected brain regions*	*Time of functional effect of mutation*
Transgenic expression of C-terminally truncated *hDISC1*	1.	Ayhan *et al.*^49^	Expression of C-terminally truncated human *DISC1* (1–598) protein leading to decreased levels of WT *Disc1*; reported dominant-negative effect	Tet-Off system: expression under condition without doxycycline; transgene induction at different time points	Mouse: mixed background (B6; SJL; CBA)	CaMKII promoter (Tet-Off; doxycycline dependent)	Expression mainly in pyramidal neurons of the forebrain and hippocampus, also in basal ganglia, amygdala, thalamus	Four groups: (1) Post and prenatal *hDISC1* expression (entire life; pre+post). (2) Prenatal expression only (until embryonic day 17; pre). (3) Postnatal expression only (from embryonic day 17; post). (4) No *hDISC1* expression (no)
	2.	Pogorelov *et al.*^50^	Expression of C-terminally truncated human *DISC1* (1–598) protein leading to decreased levels of WT *Disc1*; reported dominant-negative effect	Tet-Off system: expression under condition without doxycycline	Mouse: C57BL/6 J	CaMKII promoter (Tet-Off; doxycycline dependent)	Expression mainly in pyramidal neurons of the forebrain and hippocampus, also in basal ganglia, amygdala, thalamus	—
	3.	Niwa *et al.*^51^	Expression of C-terminally truncated human *DISC1* (1–598) protein leading to decreased levels of WT *Disc1*; reported dominant-negative effect	—	Mouse: C57BL/6	PrP promoter	Expressed widely in the brain (including cortex, striatum, NAc, hippocampus)	—
	4.	Jaaro-Peled *et al.*^52^	C-terminally truncated human *DISC1* (1–598) protein forming a dimer with WT protein leading to abnormal function and subcellular distribution; reported dominant-negative effect	Dominant-negative *DISC1* model expressed under the control of the CaMKII promoter. Two lines of DN-*DISC1* transgenic male mice: homozygous and heterozygous line 37 (higher transgene expression compared to line 10) and heterozygous line 10	Mouse: C57BL/6 N	CaMKII promoter (Tet-Off; doxycycline dependent)	Expression mainly in pyramidal neurons of the forebrain and hippocampus, also in basal ganglia, amygdala, thalamus	—
	5.	Ma *et al.*^53^	Expression of C-terminally truncated human *DISC1* (1–598) protein leading to decreased levels of WT *Disc1*; reported dominant-negative effect	—	Mouse: C57BL/6J	GFAP promoter	Astrocytes	—
*Disc1* haploinsufficiency/silencing	6.	Niwa *et al.*^54^	Transient knockdown of *Disc1* (spatially restricted, bilateral)	*In utero* injection of *Disc1* short-hairpin RNA	Mouse: ICR	H1 promoter	Pyramidal neurons of the prefrontal cortex	Pre- and perinatal stages (E14 up to minimum P7)
	7.	Kuroda *et al.*^55^	Haploinsufficiency: *Disc1*Δ2–3/Δ2–3 mice lacking exons 2 and 3 of *Disc1* gene with deficiency of full-length *Disc1* protein	Backcross generation of mutant mice	Mouse: C57BL/6JJmsSlc	Endogenous	—	—
	8.	Nakai *et al.*^56^	Haploinsufficiency: *Disc1*Δ2–3/Δ2–3 mice lacking exons 2 and 3 of *Disc1* gene with deficiency of full-length *Disc1* protein	Backcross generation of mutant mice	Mouse: C57BL/6JJmsSlc	Endogenous	—	—
Full-length *hDISC1* overexpression	9.	Vomund *et al.*^57^	Full-length human *DISC1* overexpression (spatially restricted, lateralized)	*In utero* electroporation of plasmids into rat embryos	Rat: Sprague-Dawley	CMV IE promoter	Left prefrontal cortex	Prenatal to adult stages
	10.	Trossbach *et al.*^58^	Full-length human *DISC1* overexpression leading to aggregation of DISC1	Injection of cosmid carrying the transgene into pronuclei of rats	Rat: Sprague-Dawley	Syrian Hamster PrP promoter	Expressed in all regions and cell types in the brain	—
Artificial *Disc1* mutation	11.	Lipina *et al.*^59^	Missense mutation in exon 2: T334C transition leading to a leucine to proline substitution at amino acid 100 in the *Disc1* protein (L100P)	ENU-induced artificial mutation	Mouse: C57BL/6J	Endogenous	—	—
	12.	Arime *et al.*^60^	Missense mutation in exon 2: T334C transition leading to a leucine to proline substitution at amino acid 100 in the *Disc1* protein (L100P)	ENU-induced artificial mutation	Mouse: C57BL/6J	Endogenous	—	—
	13.	Lipina *et al.*^61^	Missense mutation in exon 2: A127T transition leading to a glutamine to leucine substitution at amino acid 31 in the protein (Q31L)	ENU-induced artificial mutation	Mouse: C57BL/6J	Endogenous	—	—
Wild-type *Disc1*	14	Su *et al.*^62^	Wild-type mice	—	Mouse: C57BL/6J	Endogenous	—	—

Abbreviations: ENU, *N*-nitroso-*N*-ethylurea; *hDISC1*, human *DISC1*; GFAP promoter, glial fibrillary acidic protein promoter; GSK-3, glycogen synthase kinase-3; PDE4B, phosphodiesterase 4B—enzyme inactivating intra-cellular adenosine 3′,5′-monophosphate (cAMP); PrP, prion protein; tg*DISC1*, transgenic *DISC1*; WT, wild type.

**Table 2:** Methods

*DISC1 model category*	*Authors*^ref.^		*Animals*	n	*Gender*	*Measures*	*Brain regions*	*Technique*
Transgenic expression of C-terminally truncated *hDISC1*	1.	Ayhan *et al.*^49^	Pre+postnatal *hDISC1* (1–598) mice	6–8	Male	Locomotion in the open-field test (60 min) after amphetamine administration (1 mg kg^−1^, i.p.)	—	Behavioural analysis
			Prenatal *hDISC1* (1–598) mice					
			Postnatal *hDISC1* (1–598) mice					
			Controls					
			All groups	FC: 5–6	Male	*Post-mortem* total dopamine levels	FC, striatum, HC	HPLC-ED
				HC: 4–6	Female			
				Striatum: 4–5	Male and female			
	2.	Pogorelov *et al.*^50^	*hDISC1* (1–598) mice	8–12	Male and female	Locomotion in the open-field test (30 min) after 2 weeks treatment with non-toxic escalating dose of methamphetamine (0.5–3.0 mg kg^−1^, i.p.) vs saline administration	—	Behavioural analysis
			Controls	8–12	Male and female			
			*hDISC1* (1–598) mice	3–5	Female	Locomotion in the open-field test (10 min) 5 weeks after treatment with non-toxic escalating dose of methamphetamine (0.5–3.0 mg kg^−1^, i.p.) and a 1 mg kg^−1^ challenge dose of methamphetamine (1 mg kg^−1^)	—	Behavioural analysis
			Controls	3–5	Female			
			*hDISC1* (1–598) mice	4	Not stated	*Post-mortem* total dopamine levels after 2 weeks treatment with non-toxic escalating dose of methamphetamine (0.5–3.0 mg kg^−1^, i.p.)	FC, striatum, HC	HPLC-ED
			Controls	4	Not stated			
			*hDISC1* (1–598) mice	4	Female	Dopamine D2/3 R-binding potential in treatment naïve mice	OT, NAc, striatum, substantia nigra, VTA	[^11^C]raclopride quantitative autoradiography
			Controls	4	Female			
	3.	Niwa *et al.*^51^	*hDISC1* (1–598) mice	18–23 (9–10 male, 9–13 female)	Male and female	Locomotion after methamphetamine administration (1 mg kg^−1^, i.p.)	—	Behavioural analysis
			Isolated *hDISC1* (1–598) mice		Male and female			
			WT		Male and female			
			Isolated WT		Male and female			
			*hDISC1* (1–598) mice	6	Male	Extracellular dopamine levels after amphetamine administration (1 mg kg^−1^, i.p.)	FC, NAc	*In vivo* microdialysis
			Isolated *hDISC1* (1–598) mice	6	Male			
			WT	6	Male			
			Isolated WT	6	Male			
			*hDISC1* (1–598) mice	6	Male	Extracellular dopamine levels	FC	*In vivo* microdialysis
			Isolated *hDISC1* (1–598) mice	6	Male			
			WT	6	Male			
			Isolated WT	6	Male			
			*hDISC1* (1–598) mice	7	Male	*Post-mortem* total dopamine levels	FC, CPu	HPLC-ED
			Isolated *hDISC1* (1–598) mice	7	Male			
			WT	7	Male			
			Isolated WT	7	Male			
			*hDISC1* (1–598) mice	6	Male	D2R levels	FC, NAc	Western blot
			Isolated *hDISC1* (1–598) mice	6	Male			
			WT	6	Male			
			Isolated WT	6	Male			
			*hDISC1* (1–598) mice	6	Male	D1R levels	FC, NAc	Western blot
			Isolated *hDISC1* (1–598) mice	6	Male			
			WT	6	Male			
			Isolated WT	6	Male			
			*hDISC1* (1–598) mice	6	Male	TH levels	FC, NAc	Western blot
			Isolated *hDISC1* (1–598) mice	6	Male			
			WT	6	Male			
			Isolated WT	6	Male			
	4.	Jaaro-Peled *et al.*^52^	Heterozygous *hDISC1* (1–598) (line 37)	5	Male	Locomotion in the open field (90 min) after methamphetamine administration (1 mg kg^−1^, i.p.)	—	Behavioural analysis
			Heterozygous *hDISC1* (1–598) (line 10)	5	Male			
			Controls	6	Male			
			Heterozygous *hDISC1* (1–598) (1–598) (line 37)	5	Male	Extracellular dopamine levels after methamphetamine administration (1 mg kg^−1^, i.p.)	Ventral striatum	*In vivo* microdialysis
			Heterozygous *hDISC1* (1–598) (line 10)	5	Male			
			Controls	6	Male			
			Heterozygous *hDISC1* (1–598) (line 37)	8	Male	DAT levels	Striatum	Western blot
			Heterozygous *hDISC1* (1–598) (line 10)	15	Male			
			Controls	15	Male			
			Heterozygous *hDISC1* (1–598) (line 37)	3	Male	D2/3 R-binding potential striatum/cerebellum ratios	Striatum	[^11^C]raclopride PET
			Controls	3	Male			
			Homozygous *hDISC1* (1–598) (line 37)	6	Male	D2R-binding potential	Striatum	[^3^H]spiperone autoradiography
			Controls	5	Male			
			Homozygous *hDISC1* (1–598) (line 37)	7	Male	D2R levels	Striatum	Real-time PCR
			Controls	9	Male			
	5.	Ma *et al.*^53^	GFAP-*hDISC1* mice	13	Male	Locomotion in the open-field test (30 min) after amphetamine administration (2.5 mg kg^−1^, i.p.)	—	Behavioural analysis
				10	Female			
			Controls	15	Male			
				10	Female			
*Disc1* haploinsufficiency / silencing	6.	Niwa *et al.*^54^	*Disc1* RNAi/silencing mice	6–10	Not reported	Locomotion in the open-field test (30 min) after methamphetamine administration (1 mg kg^−1^, s.c.)	—	Behavioural analysis
			Controls	6–10	Not reported			
			*Disc1* RNAi/silencing mice	6	Not reported	Extracellular dopamine levels	mPFC	*In vivo* microdialysis
			Controls	6	Not reported			
			*Disc1* RNAi/silencing mice	7 (FC), 4 (NAc, HC)	Not reported	*post-mortem* total dopamine levels	FC, NAc, HC	HPLC-ED
			Controls	7 (FC), 4 (NAc, HC)	Not reported			
			*Disc1* RNAi/silencing mice	8	Not reported	Extracellular dopamine levels and levels after methamphetamine administration (1 mg kg^−1^, s.c.) at P56	NAc	*In vivo* microdialysis
			Controls	8	Not reported			
			*Disc1* RNAi/silencing mice	8 (mRNA), 5 (WB)	Not reported	D2R levels	mPFC	Western blot and mRNA expression
			Controls	8 (mRNA), 5 (WB)	Not reported			
			*Disc1* RNAi/silencing mice	8 (mRNA), 5 (WB)	Not reported	D1R levels	FC	Western blot and mRNA expression
			Controls	8 (mRNA), 5 (WB)	Not reported			
			*Disc1* RNAi/silencing mice	6 (IHC), 5 (WB)	Not reported	TH levels	mPFC	Western blot, immunohistochemistry
			Controls	6 (IHC), 5 (WB)	Not reported			
	7.	Kuroda *et al.*^55^	*Disc1*+/+ mice	8	Male	Locomotion in the open-field test (180 min) after methamphetamine administration (2 mg kg^−1^, i.p.)	—	Behavioural analysis
				10	Female			
			*Disc1*Δ2–3/Δ2–3 mice	10	Male			
				10	Female			
			*Disc1*+/+	7	Not stated	*post-mortem* total dopamine levels	mPFC, striatum, HC, midbrain	HPLC-ED
			*Disc1*Δ2–3/Δ2–3	9	Not stated			
	8.	Nakai *et al.*^56^	*Disc1*+/+	8	Male	Extracellular dopamine levels after amphetamine administration (2 mg kg^−1^, i.p.)	NAc	*In vivo* microdialysis
				6	Female			
			*Disc1*Δ2–3/Δ2–3	10	Male			
				6	Female			
			*Disc1*+/+	6	Male	DAT levels	NAc	Western blot
				6	Female			
			*Disc1*Δ2–3/Δ2–3	6	Male			
				6	Female			
			*Disc1*+/+	5	Male	D2R levels	mPFC, striatum, NAc, HC	Real-time PCR
				5	Female			
			*Disc1*Δ2–3/Δ2–3	5	Male			
				5	Female			
full-length *hDISC1* overexpression	9.	Vomund *et al.*^57^	Full-length *hDISC1*-overexpressing rats	11	Not stated	Locomotion in the open-field test (15 min) after amphetamine administration (0.5 mg kg^−1^, i.p.)	—	Behavioural analysis
			Control rats	10	Not stated			
	10.	Trossbach *et al.*^58^	Homozygous tg*DISC1* rats	12	Male	Locomotion in the open-field test (15 min) after d-amphetamine administration (0.5 mg kg^−1^, i.p.)	—	Behavioural analysis
			Control rats	12	Male			
			Homozygous tg*DISC1* rats	6	Male	Synaptic DAT levels	Striatum	Western blot
			Control rats	6	Male			
			Homozygous tg*DISC1* rats	6	Male	High-affinity D2^High^ receptor levels	Striatum	[^3^H]domperidone 2 nm, non-specific binding defined with 10μm S-sulpiride
			Control rats	6	Male			
			Homozygous tg*DISC1* rats	10	Male	D2/3 R-binding potential	Striatum	*In vitro* autoradiography [^3^H]raclopride
			Control rats	10	Male			
			Homozygous tg*DISC1* rats	12	Male	*post-mortem* total dopamine levels	mPFC, NAc, striatum, HC	HPLC-ED
			Control rats	12	Male			
			Homozygous tg*DISC1* rats	10	Male	D1R density	Striatum	[^3^H]SCH23390 autoradiography
			Control rats	10	Male			
Artificial *Disc1* mutation	11.	Lipina *et al.*^59^	*Disc1*-L100P mice	7–9	Male	Locomotion in the open-field test (30 min) after d-amphetamine administration (0.5, 1.0 and 1.5 mg kg^−1^, s.c.)	—	Behavioural analysis
			Controls	7–10	Male			
			*Disc1*-L100P mice	6	Male	Extracellular dopamine levels after amphetamine administration (0.5 mg kg^−1^, s.c.)	Striatum	*In vivo* microdialysis
			Controls	6	Male			
			*Disc1*-L100P mice	7	Male	*post-mortem* total dopamine levels	FC, striatum, NAc, HC	HPLC-ED
			Controls	8	Male			
			*Disc1*-L100P mice	7	Male	High-affinity D2^High^ receptor levels	Striatum	[^3^H]domperidone 2 nm, non-specific binding defined with 10μm S-sulpiride
			Controls	8	Male			
	12.	Arime *et al.*^60^	*Disc1*-L100P/L100P mice	11–12	Male	Locomotion in the open-field test (60 min) after methamphetamine administration (0.2, 0.5 or 1 mg kg^−1^, s.c.)	—	Behavioural analysis
			*Disc1*-L100P/+ mice	11–13	Male			
			+/+ mice (control)	8–9	Male			
	13.	Lipina *et al.*^61^	*Disc1*-Q31L	7	Male	*post-mortem* total dopamine levels	FC, striatum, NAc, HC	HPLC-ED
			Controls	7	Male			
Wild-type *Disc1*	14.	Su *et al.*^62^	WT+saline treated	8–12	Male	Locomotion in the open-field test (30 min) after d-amphetamine administration (1 mg kg^−1^, i.p.).	—	Behavioural analysis
			WT+TAT-D2pep	8–12	Male			
			WT+TAT-D2pep-sc	8–12	Male			

Abbreviations: Amph, amphetamine; CPu, caudate/putamen; DAT, dopamine transporter; *DISC1*D2–3/D2–3, mice lacking exons 2 and 3 of the *DISC1* gene; D2R, dopamine D2 receptor; D2/3 R, dopamine D2 and D3 receptor; FC, frontal cortex; HC, hippocampus; HPLC-ED, high-performance liquid chromatography electro-detection; i.p., intraperitoneally; KD, knockdown; Meth, methamphetamine; mPFC, medial prefrontal cortex; NAc, nucleus accumbens; OT, olfactory tubercles; RNAi, RNA interference; s.c., subcutaneously; TAT-D2pep, peptide disrupting the *Disc1*–D2R interaction; TAT-D2pep-sc, corresponding scrambled peptide; TH, tyrosine hydroxylase.

**Table 3:**
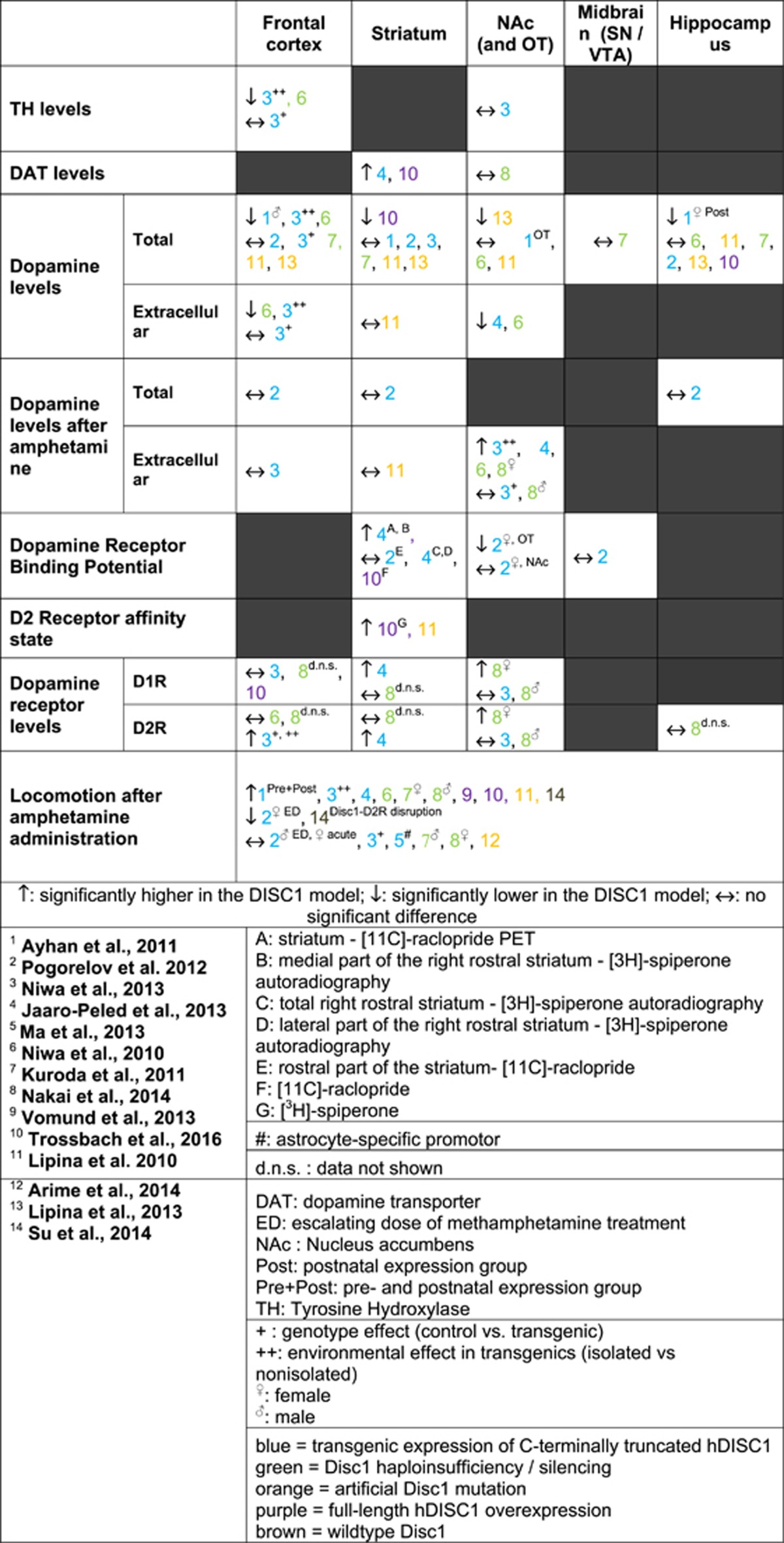
Findings
